# Effect of menstruation on school attendance of girls along with water, sanitation, and hygiene services in Northwest Ethiopia

**DOI:** 10.11604/pamj.2025.50.28.45413

**Published:** 2025-01-16

**Authors:** Yimenu Adane, Argaw Ambelu, Muluken Azage Yenesew, Yalemtsehay Mekonnen, Taddesse Kassahun

**Affiliations:** 1 Ethiopian Institute of Water Resources, Addis Ababa University, Addis Ababa, Ethiopia,; 2 School of Public Health, College of Medicine and Health Sciences, Bahir Dar University, Bahir Dar, Ethiopia,; 3 College of Natural and Computational Sciences, Addis Ababa University, Addis Ababa, Ethiopia

**Keywords:** Explore, effect, menstruation, school absenteeism, schoolgirls

## Abstract

**Introduction:**

menstruation significantly affects schoolgirls' academic attendance and performance. This study aims to explore the relationship between monthly menstruation along with the availability of water, sanitation and hygiene services, and school attendance among schoolgirls.

**Methods:**

a mixed-method cross-sectional study was conducted between March 2022 and August 2023. Study participants were selected using systematic random and purposive sampling techniques for quantitative and qualitative, respectively. A self-administered questionnaire was used to collect information from students. Focus group discussions were conducted among mothers of schoolgirls and interreligious council members, and in-depth and key informant interviews were used to gather information from parent-teacher-student association chairpersons and the vice head and head of the Education and Health department offices, respectively. Quantitative data were analyzed using SPSS version 27, and qualitative data used ATLAS.ti. The Chi-square test and binary logistic regression were used to assess the statistical significance of the relationship among variables, ensuring the thoroughness and validity of the research.

**Results:**

in this study, more than half of the schoolgirls, 389 (55.50%), missed school days due to menstruation and related factors, and on average, the schoolgirls missed 2.08 (±0.87) days per month. Factors such as WASH facilities (χ^2^= 46.78, p<0.0001; OR= 0.350, p<0,0001), accessibility with menstrual absorbent materials (χ^2^=5.42, p=0.02; OR=0.634, p=0.012), menstrual hygiene management rooms (OR=0.669, p=0.029), socio-cultural constraints (χ^2^= 22.08, p<0.0001; OR=0.539, p=<0.0001), menstrual-related pain (χ^2^=5.9, p=0.015), fear (χ^2^=36.32, p<0.0001; OR=2.505, p<0.0001), and teasing (χ^2^=13.66, p-<0.0001; OR=1.754, p<0.0001), were significantly associated with school absenteeism. A qualitative study identified four themes that influence school attendance: inadequate water and sanitation facilities, lack of access to menstrual absorbent materials, sociocultural constraints, menstrual-related pain, and fear.

**Conclusion:**

in the study area, a significant number (55.50%) of schoolgirls missed school days due to menstrual-related factors. The identified factors in the quantitative study were also supported by the themes emerging from the qualitative study. The identified modifiable factors are the areas of intervention that can increase schoolgirls´ school attendance.

## Introduction

Although menstruation is a normal physiological process that begins in girls during adolescence and is a global public health issue, it is still subjected to secrecy, shame, embarrassment, disgust, and negativity [1,2]. It has the potential to negatively impact the self-esteem and education of schoolgirls [3]. The impact of menstruation on schoolgirls´ attendance and participation in education is a significant global issue; for example, a study by Plan International revealed that in the United Kingdom, 64% of schoolgirls aged 14-21 missed part or full days of school due to menstruation [4]. Schoolgirls face several challenges relating to menstruation and managing it properly while they are in school [5]; among the challenges is the lack of affordable menstrual absorbent materials [6], which leads schoolgirls to use inappropriate and unhygienic menstrual absorbent materials [7], potentially increasing urogenital symptoms and infections, leading them to miss school days.

A lack of adequate WASH facilities in low and middle-income countries, including Ethiopia, leaves schoolgirls with limited options for healthy personal hygiene and to attend school during monthly menstruation [8]. Schoolgirls' inability to manage their monthly menstruation in schools results in school absenteeism, resulting in poor school performance, drop-out, and reduced educational attainment [7]. Poor menstrual hygiene management (MHM) of schoolgirls influences their school attendance and academic performance [9]. As a result, regular absenteeism can affect school performance, self-esteem, and ability to pursue higher levels of education [10]. Studies in different settings have reported the impact of menstruation on schoolgirls' attendance and performance [3,11,12]. School absenteeism varies from country to country [13]. For example, a study in Uganda suggested that the prevalence of school absenteeism among schoolgirls during menstruation was 59%. These schoolgirls missed one to seven school days each month [14]. Poor access to menstrual-absorbent materials and a poor school environment with inadequate water, sanitation, and hygiene (WASH) facilities can make it difficult for schoolgirls to attend school [15]. Poor MHM impacts school attendance and academic performance [6].

School absenteeism is a widespread issue that affects schoolgirls of all ages [16], with various associated factors contributing to its prevalence, such as WASH-related issues, lack of menstrual absorbent materials, and sociocultural norms [17]. Studies show that school absenteeism can significantly impact academic performance, social development, and overall well-being [18,19]. For example, the prevalence of menstruation-related school absenteeism in rural Ghana was 27.50%, ranging from one to seven days during the menstrual period [3]. In southeast Nigeria, schoolgirls´ school absenteeism was 56.50% due to pain during menstruation, which was present in 82% of the schoolgirls [20]. A study in Ethiopia found that 50% of schoolgirls miss between one and four days of school every month due to their monthly menstruation [21].

The Ethiopian government recognizes the need to invest in initiatives through the Ministry of Education (MoE) to improve MHM among schoolgirls. One of the initiatives in Ethiopia is the former President's effort to provide free menstrual absorbents to millions of schoolgirls. A directive in 2020/2021 also reduced tariffs on imported menstrual absorbents from 35% to 10%. National platforms have also been organized to celebrate the annual MHM event since 2017. However, there is no up-to-date information on schoolgirls´ school absenteeism after the above interventions have been implemented. It is important to conduct further studies to know the impact of such interventions. Moreover, the extent of school absenteeism due to menstruation and menstrual-related factors is vital to strengthen the existing strategies and devise context-based interventions. Therefore, the purpose of this study is to see the relationship between monthly menstruation along with the availability of water, sanitation and hygiene services, and school attendance among schoolgirls.

## Methods

**Study area:** the study was conducted in five primary and five secondary schools in the Bahir Dar City administration: six secondary schools were in urban areas, and four primary schools were in the city's satellite-urban areas. Bahir Dar is the capital of the Amhara region in Ethiopia, located at 11° 14´ 60.00” N, 37° 09´ 60.00” E, on the southern shore of Lake Tana. The city is located 565 km northwest of Addis Ababa. Studies focusing specifically on the effects of menstruation, along with water, sanitation, and hygiene, in such poor settings are limited. There have been no studies highlighting how menstruation and WASH services impact school attendance and education for schoolgirls in the study area. Conducting studies in this area will help to advocate for better information and education on menstrual health in schools, ultimately helping to reduce school absenteeism among schoolgirls. As per 2020 city administration statistics, the city administration has a total of 52 schools, 41 primaries (1-8) and 11 secondary (9-12) schools. A total of 65,359 students were enrolled in grades 1-12^th^; from these, 33,673 were female students. Though the WHO and UNICEF Joint Monitoring established the JMP ladder for WASH services as advanced, basic, limited, and no services, these services in school settings continued a significant challenge; hence, the JMP 2022 report indicated that 15%, 13%, and 25% of schools globally, and in Ethiopia 76%, 39%, and 80% schools had no basic water, sanitation, and hygiene services respectively [22].

**Study design:** a mixed methods approach was used to triangulate contextual insights from qualitative data with broadly applicable findings from quantitative data. The data presented in this paper are from mixed-method studies that include cross-sectional surveys, focus group discussions (FGDs), in-depth interviews (IDIs)), and Key Informant Interviews (KIIs). The study commenced with collecting data via a self-administered questionnaire, followed by qualitative interviews, to provide a comprehensive overview and a deeper understanding of the effect of menstruation on schoolgirls in the Bahjr Dar city administration. Considering the sensitive nature of the topic, mixed methods were found to be valuable in exploring its effect.

**Study population:** the study population included schoolgirls enrolled in primary and secondary schools during the 2021/22 academic year in Bahir Dar city. Mothers, parent-teacher-student association chairpersons (PTSAs), interreligious council members (IRC), and vice head of Education and Head of Health offices of Bahir Dar City were also study populations for qualitative study.

**Inclusion criteria:** the quantitative study involved schoolgirls aged 11 to 25 years who had menstruated at least twice and consented to participate after being informed of the study's objectives and expressing interest. schoolgirls who had not started menstruating or had started menstruating but were unwilling to participate or were absent from school during the data collection days were not included in the study. The qualitative study in FGDs, IDIs, and KIIs included all who consented to participate after being informed of the study's objectives and expressing interest.

**Sample size:** to determine the number of schoolgirls to be included in the quantitative study, the sample size was calculated using a single population formula [13,14]. The proportion of the desired outcome (p) was taken as 50% since there was no prior national or local data on the effect of menstruation among schoolgirls in a similar setting. A non-response rate of 15% was anticipated due to the sensitivity and stigma surrounding menstruation. Many schoolgirls may feel uncomfortable discussing personal and private aspects of their menstrual hygiene, leading to reluctance to participate. In addition, participants may fear judgment or scrutiny based on their responses, and a lack of awareness or knowledge about the subject may contribute to non-response. Plugging the above values into the sample size determination formula in [23] and [24], a sample size (n) of 705. For the qualitative study, 23 mothers, three PTSA chairpersons, three IRC members, the vice head, and the head of the Bahir Dar sub-city education and Health departments were considered.

**Cross-sectional survey data collection:** a self-administered, pre-tested questionnaire was used among 701 primary and secondary schoolgirls in the Bahir Dar city administration. Data were collected on societal behaviors, missed school days in the last month due to menstruation, and commonly reported reasons for schoolgirls' absenteeism. The research team members reviewed the completeness and consistency of the data at the end of each data collection day.

**Qualitative data collection:** interviews were conducted in the local language, using semi-structured interview guides containing open-ended questions and suggested probing questions. The interviewer was a female public health professional and a PhD candidate. FGDs consisted of purposively selected mothers of schoolgirls who attended the self-administered questions for the cross-sectional survey and IRC members of the Bahir Dar city administration. IDIs consisted of the PTSA chairpersons, and KIIs consisted of the vice head and head of the Education and Health Department Offices, respectively. The discussion covered topics such as knowledge and attitudes towards menstruation, cultural norms associated with menstruation, menstrual absorbent materials and usage, challenges faced in managing monthly menstruation, opinions on school WASH facilities, including MHM rooms, reasons for school absenteeism during menstruation or dropping out of school, the role of parents and school communities, the government and partners in supporting and providing potential solutions for MHM for schoolgirls while they menstruate. Interviews were audio-recorded, transcribed, and translated into English. Field notes were also taken. Data were then imported into ATLAS.ti software (Version 7.5,18), ATLAS.ti Scientific Software Development Mnbh, Berlin) for analysis. Each interview lasted, on average, 40 to 50 minutes. Because of the sensitive nature of the topic, the moderator allocated some time at the beginning of the discussions. Data saturation was used as an indicator of sufficient sampling [25].

### Data analysis

**Quantitative data:** descriptive statistical, chi-square tests and binary logistic regression were used to describe the study variables and examine associations. The Statistical Package for Social Science version 27 was used for data analysis, and a p-value of 0.05 was considered as a cut-off point for statistical significance.

**Qualitative data:** the field team simultaneously translated and transcribed data from the FGDs, IDIs, and KIIs into English. Each researcher assigned meaningful segments a code, then discussed and compared. ATLAS.ti software (Version 7.5,18) was used to analyze the qualitative study.

**Ethics approval and consent to participate:** this research was conducted according to the ethical principles of the Declaration of Helsinki. All participants provided informed consent before their inclusion in the study. Ethical clearance was obtained from the Research Ethics Committee of the Ministry of Education (Ref. No.712-219/m259/35). Data were collected after getting written consent from the schoolgirls, and for those below 18 years, permission from the school administration was sought, giving the school principal a form to be signed. Before administrating the questionnaire, schoolgirls under 18 who participated in the study were informed about the research and their right to participate or not to participate in the study. Parents/guardians were contacted through the school principals and gender club leaders, who explained and distributed information sheets about the study and consent forms to their students to take home and inform their parents/guardians. The consent of girls below 18 years was obtained from their families/ guardians. Additionally, the school’s consent was obtained before administering the questionnaire to the schoolgirls.

## Results

**Characteristics of the study participants:** a total of 701 schoolgirls participated in the study, which led to a response rate of 99.43%. Most participants, 413 (58.90%), were urban residents. The data reveal that the study predominantly involved older schoolgirls (grades 9-11) (Table 1). A total of 23 mothers, three IRC members of Bahir Dar City, three PTSA chairpersons, the Bahir Dar City Health Department Head, and the Bahir Dar City Education vice head participated in the qualitative study (Table 2).

**Table 1:** characteristics of the participants in the quantitative study

Variables	Frequency	%
**Grade level of schoolgirls**		
7-8	122	17.40
9-11	579	82.60
**Residence of schoolgirls**		
Urban	413	58.90
Urban-satellite	288	41.50

**Table 2:** number of discussions and participants in the qualitative study

Tools	Number of groups	Participants	Number of participants
FGDs	3	Mothers	23
FGD	1	Interreligious council members	3
IDIs	-	Parent-teacher student association chairpersons	3
KIIs	-	Head of Bahir Dar Health Office	1
		Vice Head of Bahir Dar Education Office	1

FGD: focus group discussion; IDIs: in-depth interviews; KIIs: key informant interviews (KIIs)

### Findings from a quantitative study

**Information about missed school days during monthly menstruation:** from the total number of schoolgirls who missed school days, on average, schoolgirls missed 2.08 (±0.87) schooldays monthly due to different factors associated with menstruation. Table 3 depicts monthly school absenteeism due to menstruation. A total of 312 schoolgirls, accounting for 44.50%, reported being absent for 0 days. In contrast, 108 (15.40%) schoolgirls were absent for one day, while 166 schoolgirls, representing 23.70%, missed two days. Ninety-one schoolgirls, or 13%, were absent for three days, and a smaller group of 24 (3.40%) schoolgirls missed four days. Overall, 389 (55.50%) schoolgirls missed one to four school days. The most prominent factor for being absent from schools during menstruation, affecting approximately 264 (67.90%) of the schoolgirls, was lack of access to safe Menstrual Hygiene Management (MHM) rooms. Moreover, 255 (65.60%) of the schoolgirls mentioned inaccessibility to MHM materials as a key reason for their absenteeism. Menstrual-related pain, sociocultural constraints, and fear also play substantial roles, with 270 (69.40%), 282 (72.50%), and 207 (53.20%) of schoolgirls, respectively, identifying these as reasons for missing school. Discomfort, reported by 254 (65.30%) of schoolgirls, further compounds the issue. Inadequate WASH facilities were a concern for 219 (56.30%) of the respondents, while teasing from classmates affected 234 (60.20%).

**Table 3:** number of missed school days and reasons for missing the school days

Variables	Frequency	%
**Number of schoolgirls who missed school days**		
One day	108	15.40
Two days	166	23.70
Three days	91	13.00
Four days	24	3.40
Mean (±SD)	2.08 (±0.87)	
**Reasons for missed schools**		
Lack of MHM room	264	67. 90
Inaccessibility to menstrual absorbent materials	255	65.60
Menstrual related pain	270	69.40
Socio-cultural constraints	282	72.50
Fear	207	53.20
Discomfort	254	65.30
Inadequate WASH facilities	219	56.30
Teasing from classmates	234	60.20

MHM: menstrual hygiene management; WASH: water, sanitation, and hygiene

### Factors associated with menstrual-related school absenteeism

**WASH facilities:** this study revealed that from a total of 389 participants who missed school days, only 170 (43.70%) reported the presence of adequate WASH facilities in their schools; however, a larger proportion, 219 (56.30%) of schoolgirls who missed school days reported having inadequate WASH facilities. There appears to be a statistically significant association (χ^2^= 46.78, p-value < 0.0001) between the availability of WASH facilities and school attendance, suggesting inadequate WASH facilities may contribute to absenteeism. The result from a binary logistic regression revealed that having access to adequate WASH facilities lowers the likelihood of missing school by 35% (OR= 0.350) when the other contributing factors are held constant. The p-value of <0.0001 indicates that this result is statistically significant, emphasizing the critical role of proper WASH facilities in reducing menstruation-related absenteeism. When schools provide clean, private, and accessible WASH facilities for menstrual hygiene management, it not only supports the health and well-being of students but also enables them to attend school without the barriers posed by inadequate WASH facilities.

**Accessibility to menstrual absorbent products:** in the study area, schoolgirls were not able to access menstrual-absorbent materials as needed. Of the study participants who missed school days, only about 134 (34.40%) reported regular access to menstrual absorbent products in school settings. In contrast, a larger proportion of schoolgirls who missed classes, 255 (65.60%), reported having inadequate menstrual absorbents. There is a statistically significant association (χ^2^= 5.42, p-value= 0.02) between the availability of menstrual absorbent materials and school attendance, indicating menstrual absorbent materials may contribute to absenteeism and having adequate menstrual absorbent materials in school decreases the odds of missing school by 36.6% (OR= 0.634) as demonstrated in binary logistic regression. The p-value of 0.012 indicates statistical significance, meaning that access to adequate materials reduces the likelihood of absenteeism.

**Access to MHM rooms:** the majority of schoolgirls, 255 (65.60%), who missed classes reported inadequate MHM rooms in their schools as the reason for school absenteeism. The study informed that access to a safe Menstrual Hygiene Management (MHM) room lowers the likelihood of missing school by 33.1% (OR= 0.669). The p-value of 0.029 indicates that this finding is statistically significant, highlighting the importance of having a designated, safe space for managing menstruation as a key factor in promoting school attendance.

**Social and cultural constraints:** the study revealed that the majority, 282 (72.50) of schoolgirls who missed classes reported having socio-cultural constraints, with a statistically significant association (χ^2^= 22.08, p-value < 0.0001) between the impact of sociocultural constraints and school attendance. The result from binary logistic regression suggests that sociocultural factors that influence school attendance during menstruation are associated with a 46.10% reduction in the likelihood of missing school (OR= 0.539). The p-value of <0.0001 confirms that this result is highly statistically significant, suggesting that good societal norms and cultural expectations and avoiding stigma related to menstruation play a major role in reducing school absenteeism.

**Menstrual-related pain, fear, and discomfort:** pain related to menstruation causes substantial anxiety in schoolgirls in study areas; hence, a larger proportion of girls who missed classes 270 (69.40%) reported having menstrual-related pain. There is a statistically significant association (χ^2^= 5.97, p-value= 0.015) between the feeling of menstrual pain and school attendance, which suggests that menstrual pain may contribute to absenteeism. Although the odds of missing school are reduced by 23.5% (OR= 0.765), this result is not statistically significant (p-value= 0.124), suggesting that, when accounting for other factors, menstrual pain may not be a major predictor of absenteeism. Most schoolgirls, 207 (53.20%), who missed classes reported feelings of fear, in which there is a statistically significant association (χ^2^=36.32, p-value<0.0001) between fear and school attendance. Fear related to menstruation, such as feelings of embarrassment or anxiety, significantly increases the likelihood of missing school by 150.5% (OR = 2.505). The p-value of <0.0001 indicates that fear associated with menstruation is a powerful predictor of school absenteeism. This suggests that when schoolgirls experience emotional distress, such as fear of being stigmatized during menstruation, it can substantially affect their willingness or ability to attend school.

Although the majority of schoolgirls, 254 (65.30%), who missed classes reported discomfort, there is no statistically significant association (χ^2^=2.66, p-value=0.103), suggesting that discomfort may not contribute to absenteeism and even though the odds of missing school are reduced by 22.8% (OR=0.772); still, this result does not reach significance at the 0.05 level, indicating that discomfort alone does not seem to be a strong predictor of absenteeism. Most schoolgirls, 234 (60.20%), who missed classes reported teasing from school communities, especially from classmates, was the reason for school absenteeism, with a statistically significant association (χ^2^=13.66, p-value p<0.0001). Teasing during menstruation increases the odds of missing school by 75.4% (OR= 1.754). With a p-value of <0.001, this result is statistically significant, highlighting that menstruation-related teasing is a strong contributor to absenteeism. Generally, the findings in Table 4, Table 5 indicate the factors associated with school absenteeism.

**Table 4:** factors associated with menstruation-related school absenteeism (n= 701)

Variable	Category	A schoolgirl missed classes		Chi-square	p-value
		No (column %)	Yes (column %)		
Availability of menstrual absorbent materials in school setting	Not adequate	230 (73.7)	255 (65.6)	5.42	0.02*
	Adequate	82 (26.3)	134 (34.4)		
Safe MHM room	No	228 (73.1)	264 (67.9)	2.27	
	Yes	84 (26.9)	125 (32.1)		
Sociocultural behavior impacts school attendance during menstruation	No	139 (44.6)	107 (27.5)	22.08	0.000*
	Yes	173 (55.4)	282 (72.5)		
Inadequate WASH facilities in the school impact the management of menstruation	No	217 (69.6)	170 (43.7)	46.78	0.000*
	Yes	95 (30.4)	219 (56.3)		
Pain during monthly menstruation	No	123 (39.4)	119 (30.6)	5.97	0.015*
	Yes	189 (60.6)	270 (69.4)		
Fear during monthly menstruation	No	77 (24.7)	182 (46.8)	36.32	0.000*
	Yes	235 (75.3)	207 (53.2)		
Discomfort during menstruation	No	127 (40.7)	135 (34.7)	2.66	0.103
	Yes	185 (59.3)	254 (65.3)		
Teasing from school communities during menstruation	No	168 (53.8)	155 (39.8)	13.66	0.000*
	Yes	144 (46.2)	234 (60.2)		

* Significant at 0.05 level of significance

**Table 5:** binary logistic regression output for factors associated with menstruation-related school absenteeism (n= 701)

Variable	Category	Coefficient	Std. Error	Adjusted Odds Ratio (OR)	95% CI for OR	p-value
Constant		1.773	0.276	5.886		0.001*
Availability of menstrual absorbent materials in school setting	Not adequate (Reference)					
	Adequate	-0.456	0.182	0.634	(0.443, 0.906)	0.012*
Safe MHM room	No (Reference)					
	Yes	-0.402	0.184	0.669	(0.467, 0.960)	0.029*
Sociocultural behavior impacts school attendance during menstruation	No (Reference)					
	Yes	-0.618	0.173	0.539	(0.384, 0.757)	0.000*
Adequate WASH facilities in the school to manage monthly menstruation	No (Reference)					
	Yes	-1.051	0.170	0.350	(0.251, 0.488)	0.000*
Pain during monthly menstruation	No (Reference)					
	Yes	-0.267	0.174	0.765	(0.544, 1.076)	0.124
Fear during monthly menstruation	No (Reference)					
	Yes	0.918	0.178	2.505	(1.768, 3.547)	0.000*
Discomfort during menstruation	No (Reference)					
	Yes	-0.258	0.173	0.772	(0.551, 1.083)	0.134
Teasing from school communities during menstruation	No (Reference)					
	0.562	-0.562	0.167	0.570	(0.411, 0.791)	0.001*

* Significant at 0.05 level of significance; MHM: menstrual hygiene management; WASH: water, sanitation, and hygiene

**Findings from a qualitative study:** twenty-three mothers whose daughters had experienced menarche participated in FGDs along with three members of the IRC members. Additionally, three chairpersons from the PTSA took part in IDIs, and KIIs were conducted with the vice head of education and head of Health offices (Table 2). In a qualitative study, four themes emerged as driving factors for missing school days. These themes include inadequate WASH facilities, lack of access to menstrual hygiene products, socio-cultural constraints, and menstrual-related pain and discomfort (Table 6).

**Table 6:** main and sub-themes from a qualitative study

SN	Main theme	Sub-theme
1	Lack of access to menstrual absorbent products	Families' economic challenges in providing menstrual absorbent products
		Lack of available menstrual absorbent products in schools
		Insufficient ownership and responsibility of the school community to avail menstrual absorbent products in schools
2	Inadequate WASH facilities	Lack of adequate and safe water supply in schools
		Lack of segregated toilet facilities in schools
		Lack of handwashing facilities in schools
3	Socio-cultural constraints	Restricted activities, going to religious places, attending social gatherings, and touching religious materials like holy books.
		Gossip, teasing
		Advice not to attend social gatherings during menstruation and not to go to school.
		Negative community perceptions and naming of the menstruation
4	Pain and fear	Menstrual pain and its impact on attendance
		Anxiety, fear, and stress during menstruation
		Isolation, depression, and discomfort

**Theme one: inadequate WASH facilities:** it was indicated that schoolgirls who attended schools with inadequate school WASH facilities preferred to stay at home during their monthly menstruation, which two mothers in FGDs said. *In school settings, it is challenging for schoolgirls to practice proper menstrual hygiene management due to inadequate WASH facilities. As a result, schoolgirls often use the same menstrual absorbent material for an entire day because of the lack of WASH facilities to change it. This situation leads schoolgirls to stay home and miss school days. Hence, their home is preferable to wash and change used menstrual absorbent materials*(FGD2,05, FGD3, 06). One mother supported this statement by saying that *schoolgirls who used to perform best grades were declining after their first menarche, mainly due to inadequate WASH and MHM facilities. They miss school days every month*(FGD4,05). The contribution of inadequate WASH facilities in school settings to missing school days was witnessed by interreligious council members (FGD4,03), the PTA chairperson (IDIs 02), the vice head of Education Office, and the Head of the Health Office in qualitative interviews (KIIs 01 and 02).

**Theme two: inaccessibility to menstrual absorbent materials:** in the FGDs made with mothers and IRC members, most participants witnessed the challenges schools face in providing adequate and safe menstrual absorbent materials to schoolgirls during menstruation. For example, one mother in FGDs informed that *the lack of menstrual-absorbent materials leads schoolgirls to miss school days. She added that my daughter started menstruating at the age of Nine, and she informed me that the school is not providing it* (FGD2,08). This idea was supported by an IRC member who said that *due to a lack of menstrual absorbent materials, schoolgirls missed more school days than boy students* (FGD4,03). One PTSA chairperson said *the impact is more significant when schoolgirls are without menstrual absorbent materials. Menstruation threatens the schoolgirls to learn equally with boy students* (IDI 01). In KIIs, the vice head of the Bahir Dar City Education Office has said that *we cannot support all menstruating schoolgirls, but when we get support from partners, we supply only for schoolgirls who are facing economic problems* (KIIs 01). In contrast, one member of the IRC argued that menstruation is not a challenge in urban settings. He stated that *menstruating women and schoolgirls in urban areas can access modern menstrual absorbent materials and do not encounter any problems related to menstruation* (FGD4, 02).

**Theme three: socio-cultural constraints hindering menstrual hygiene management:** in FGDs, IDIs, and KIIs, all participants spoke of many socio-cultural constraints schoolgirls face during menstruation; almost all IRC members agreed that *due to the sociocultural constraints within the community, some boy students considered menstruation as fun and teased when they (boy students) realize blood stain on schoolgirls uniform, at the moment schoolgirls feel ashamed, and fear; as a result, they miss school days* (FGD4, 01,02, and 03). He again said *I used to be a teacher in a primary school (Grade 8); at that time, schoolgirls mostly missed schooldays due to monthly menstruation. The issue is more significant in rural schools due to shame and fear. In contrast, in urban schools, the problem is not significant except menstrual-related pain and discomfort but will be improved after marriage, especially after she gives birth* (FGD4, 01). Mothers in FGDs also strengthened the impact of socio-cultural constraints by saying. *There is no open discussion within families and school communities due to culture and taboos related to menstruation; when the blood stain is seen, classmates tease, and schoolgirls feel ashamed, then they leave class and go back home; the majority miss school days until the end of the menstruation* (FGD2,08). One PTSA chairperson also supported this by saying *teasing from boy students due to cultural issues made schoolgirls feel ashamed and fearful and resulted in missing school days* (IDI 01). The Vice Head of Bahir Dar Education Office agreed *by saying yes that menstruation and poor MHM have an impact; for example, due to a lack of knowledge on menstruation, teasing from boy students has a negative impact on schoolgirls* (KIIs 01).

**Theme four: menstrual-related pain and fear:** participants in all types of qualitative studies agreed that pain, fear, and discomfort were the primary reasons for school absenteeism. For instance, mothers, IRC members, and PTSA chairpersons all provided similar feedback. For example, a mother in a focus group discussion stated that: *During menstruation, my daughter usually has abdominal pain; when I advised her to drink hot things like tea, she believed drinking hot fluid like tea would increase the flow of blood, and due to the absence of a safe and appropriate place to change the used materials, she missed schooldays*(FGD2,03). Interreligious council members (IRC) member also explained about menstrual-related pain by saying that *for some schoolgirls, menstrual-related pain requires treatment, so we advise them to take medication; for those who face a challenge during monthly menstruation, I recommend to them to stay in their home to care for themselves by absenting themselves from the school*(FGD4, 03).

## Discussion

This study investigated how menstruation affects school attendance among schoolgirls and assessed the prevalence of missed school days and factors for menstrual-related school absenteeism. schoolgirls face challenges in practicing proper MHM during their monthly menstruation; due to this, about 20% of schoolgirls miss school days globally, and one in 10 will drop out entirely [26]. Menstruation significantly affects schoolgirls´ academic performance and is one of the leading causes of their short-term absences from school [27]. The findings highlight the challenges schoolgirls face in managing their menstruation alongside schooling. Missing school days means losing critical lessons in class and, sometimes, class and homework assignments, including examinations, which may never be recovered after they return to school [3]. This study showed that the factors related to menstruation in schools of Bahir Dar city are a real challenge and are associated with missed school days. Both quantitative and qualitative findings show similar factors for school missing.

The number of schoolgirls who missed school days 389 (55.50%) in this study is higher than the findings from Urban slum of Madhya Pradesh (50.60%) [28] Niamey, Niger (15%) [29], Burkina Faso (17%) [29], Uganda (19.70%) [14], Nigeria (23%) [29], rural Gambia (27%) [10], rural Northern Gahanna (27.50%) [3], Lao PDR (29.70%) [30], Delhi India (40%) [31], Bangladesh (41%) [32], Nepal (46.70%) [33], Tanzania (47%) [34] and from a pooled result from a systematic review and meta-analysis in Ethiopia (32.03%) [7], in Cross River State, Nigeria (28%) [14,35], and in Lao PDR (26.50%) [30]. However, the number of schoolgirls who missed school days in this study is lower than the findings from Nepal (93%,61.40%) [36,37], Southeast Nigeria (56.50%) [20], and in urban areas in Northeastern Ethiopia (79%) [38], in Oromia region of Ethiopia (57.70%) [39]. This study somehow resembles the findings from North East Ethiopia (54.51%) [40]. Furthermore, it is projected that one out of every ten African school-aged schoolgirls misses four days every month due to menstruation-related driving factors [14]. Similar challenges in school attendance were reported in various studies in Africa, the Middle East, Asia [30,31], and Bangladesh [41]. This makes schoolgirls disadvantageous to male students and potentially impacts their academic performance [31].

The reason for the differences in the days of missing school days across the countries could be a range of contextual differences, notably varying levels of access to menstrual absorbent materials, WASH facilities, and the economic status of the families that may influence the purchase of menstrual absorbent materials, cultural beliefs and stigma surrounding menstruation, level of education and awareness about MHM, and the study methodology in which different studies might use varying methods leading to different results [9]. Inadequate school WASH facilities, inaccessibility to menstruation-absorbent materials, socio-cultural constraints, teasing from classmates, menstrual-related pain, and fear of stains and discomfort were identified as factors for menstruation-related missed school days (Table 4, Table 5). The findings of this study were supported by previous studies made in low-and middle-income countries (LMICS) [3,15,29,42]. For example, in school settings, a lack of clean, private, and gender-specific WASH facilities [2], a lack of menstrual absorbent materials (sanitary pads) [3,11,31], cultural restrictions [26,43-46], menstrual pain [6,32], and fear of being teased at school [3] have been reported to be associated with school absenteeism. Qualitative studies have also supported the fact that schoolgirls often miss school days during menstruation due to inadequate school WASH facilities, inaccessibility to menstrual absorbent materials, socio-cultural constraints, pain, and fear related to mensuration [47]. These findings have been consistent across different studies, as reported in FGDs, IDIs, and KIIs [47-49].

**WASH facilities:** accessing to school WASH facilities influences schoolgirls' attendance and educational achievements [50]. schoolgirls in Ethiopia face challenges due to inadequate and poor school WASH facilities (lack of access to consistent supplies of water, lack of access to segregated and friendly toilets, and hand hygiene facilities); as a result, schoolgirls reported frequent episodes of low school attendance and missing school days [51]. Providing safe, private WASH facilities for changing menstrual materials, washing and drying materials, discrete disposal options, and soap to maintain personal hygiene are important factors in reducing the number of missed schools. When schools lack adequate WASH facilities for proper MHM practices, schoolgirls often miss school days during their monthly menstruation [5]. In this study, more than half (55.20%) of participants in the quantitative study reported that inadequate school WASH facilities in their schools affected their school attendance during menstruation. From those who reported the presence of inadequate WASH facilities in school settings, about 56.30% of schoolgirls missed one to four schooldays during menstruation. The inadequate WASH facilities were significantly associated with missed school days. The finding is similar to the baseline study of UNICEF, Ethiopia [52]. A systematic review on menstruation in Nepal has also indicated that inadequate WASH facilities in schools lead to school absenteeism, which is also similar to the findings conducted in different studies as well [53] but lower than (76.70%) from India [31]. In the qualitative study, mothers in FGDs and IRC members reported that inadequate school WASH facilities have caused schoolgirls to miss several days each month. However, in the KIIs, KII 02 has reported that all schools possess WASH facilities, regardless of the quality. Again, KIIs 02 argues that WASH challenges are universal in community and school settings. The reality is that in schools of developing nations like Ethiopia, specifically in study areas, accessing adequate and safe WASH facilities is a challenge and the main factor for schoolgirls to miss school days each month.

**Accessibility to menstrual absorbent materials:** to prevent physical discomfort and leakages, schoolgirls need safe and adequate menstrual-absorbent materials [9,54]. In this study, menstrual-related missed school days were significantly associated with inaccessibility to menstrual-absorbent materials. The finding was similar to the findings made in other countries that reported the association of school absenteeism with the inaccessibility of menstrual-absorbent materials [10,20,41]. In Ethiopian schools, most schoolgirls face a challenge in accessing menstrual-absorbent materials; hence, those who did not use mensural-absorbent materials were more likely to be absent from school [55]. In the study, of (69.20%) who reported inaccessibility of menstrual absorbent materials, 65.60% of schoolgirls missed one to four school days (Table 3). The findings are lower than the findings from Ghana (82.20%) [9] and higher than the findings from Nigeria (51.10%) [56]. However, studies conducted in Tanzania have reported no significant association between access to menstrual-absorbent materials and school absenteeism [44].

In qualitative studies, most mothers and IRC members in the FGDs and officials in the KIIs reasoned out that the inaccessibility of menstrual-absorbent materials was the main reason and factor for schoolgirls missing school days. However, one participant in the focus group discussions believed that access to menstrual absorbent materials was not challenging for schoolgirls and women in urban areas, as they could easily obtain them. This is the opinion of the participant, but in the study area, the situation could be a challenge, as stated by (FGD4 O2). In the KIIs, officials from the Bahir Dar City Health and Education Department reported that some partners working in the WASH program used to supply some packets of menstrual absorbent materials to schools. However, the initiative was partner-dependent and did not ensure its sustainability. During the interviews, participants cited several main reasons for the inaccessibility of menstrual absorbent materials. These include the economic challenges families face in providing these products, the absence of these products in schools, and insufficient ownership and responsibility within the school community to provide menstrual absorbent products. This may be due to the differences in socio-cultural awareness and commitment of the school community, WASH sectors, and families to providing menstrual absorbent materials to schoolgirls.

**Socio-cultural constraints:** poor MHM, because of socio-cultural constraints, has been shown to result in a sense of shame, anxiety, fear, and embarrassment that contributes to missing school and poor performance at school. This study considers sociocultural constraints as norms or interpersonal interactions within communities that can prevent schoolgirls from school days during their menstruation [44]. Evidence shows that schoolgirls report feelings of discomfort, fear, and shame related to menstruation [56]. The prevalence of menstruation-related missed school days in this study was more common among schoolgirls who were culturally restricted during menstruation; hence, fear, teasing, and shame were factors that prevented schoolgirls from understanding the scientific facts and the naturalness of menstruation. The finding of this study confirmed that of the total of 701 schoolgirls who attended the quantitative study, 72.50% claimed they face challenges related with socio-cultural constraints during menstruation, particularly 53.20% missed due to fear of being seen and 60.20% missed due to teasing, which were the main factors for missed school days. Fear of schoolgirls because of teasing may cause schoolgirls to feel shame and discomfort, and as a result, it can deteriorate schoolgirls' capacity for education quality and reinforce them to miss school days [57].

Qualitative findings complemented the quantitative findings from this study; as a result, the FGDS, IDIs, and KIIs participants have confirmed that the existing sociocultural constraints, such as not allowing menstruating individuals, including schoolgirls, to attend religious ceremonies and social gatherings, touching holy books, drinking hot fluids like tea exacerbate menstruation flow, marriage and giving birth relief pain related with menstruation and sometimes not allowing schoolgirls to go to school leading them to feel shame and fear were the main reason for schoolgirls to miss school days in the study area. Many qualitative studies have documented the existence of explicit socio-cultural restrictions on menstruating schoolgirls and women [58]. Evidence suggests that menstrual-related teasing is a common experience among schoolgirls, which results in their school participation and attendance [43,45]. Thus, Socio-cultural constraints such as fear, teasing, and discomfort surrounding menstruation often prevent schoolgirls from attending, participating, and concentrating in class [59].

**Menstrual related pain:** menstrual-related pain was the most common reason for schoolgirls to miss school days during their menstruation. According to a study, it is the main reason for school absenteeism [10] and is frequently identified as the reason for school absenteeism in high, low, and middle-income countries [60]. Quantitative findings in this study confirmed that menstrual-related pain was the main reason for 69.40% of schoolgirls missing school days, a finding which is higher than the study from three states of India (4.70%) [15] and lower than, (76.30%) [31], (76.00%) [14], (82.20%) [9] from the study reported in Delhi, India, Uganda, and from the rural community of Ghana respectively. In this study, even though about 69.40% of schoolgirls missed school days due to menstrual-related pain, menstrual-related pain was not significantly associated with school absenteeism. However, a study made in Uganda reported that missing school days are associated with menstrual-related pain [14]. In the qualitative interviews, mothers and IRC members in FGDs stated that menstrual-related pain was the reason for missing school days. However, IRC members in FGDs (FGD4,03) believed that marriage and giving birth would resolve pain related to it; such assumptions and thoughts may need further scientific studies. One mother (FGD2,03) advised her daughter to drink hot fluids to relieve menstrual pain. However, her daughter believed that hot water would increase the flow and amount of menstruation. Such thoughts and beliefs pose challenges for schoolgirls during their monthly menstruation. Generally, challenges encountered by schoolgirls in Ethiopia regarding school absenteeism during menstruation may hamper realizing the sustainable development goal (SDG4) to ensure inclusive and equitable quality education and promote lifelong learning opportunities for all.

**Limitations of the study:** in the quantitative study, it is important to note that the study focused on schoolgirls in grades 7-11, potentially overlooking younger schoolgirls in lower grades who have recently started menstruating. As the study used self-administered questions, schoolgirls may have limited skills to understand and respond accurately. The subject of menstruation is taboo in the study area, so they may feel uncomfortable answering the questions. Additionally, girls may have difficulty recalling specific details about MHM practices. The self-administered questions may not capture the full context of the MHM practices.

## Conclusion

The study revealed that the effect of menstruation on school attendance was high in the study area, which is more than half of schoolgirls, 389 (55.50%), missed school days due to menstruation and related factors; the findings also emphasize the significant impact of menstruation and inadequate WASH services on school absenteeism. Schools lack a conducive and supportive environment for schoolgirls during their menstruation. The finding also highlights that there are socio-cultural constraints on menstruation, inadequate school WASH facilities, a lack of menstrual-absorbent materials, teasing from classmates, and fear of blood stains and pain. These factors were the primary cause for schoolgirls to miss school days and are significantly associated with school absenteeism. To address these challenges, it is essential to improve WASH facilities in schools and ensure access to menstrual products. This will help reduce absenteeism among schoolgirls, which is crucial for promoting gender equality and enhancing educational outcomes in the study area. Therefore, schoolgirls should have training on menstruation and menstrual hygiene management via the school curriculum. School management, including teachers, should have specific information about menstruation and should be able to support menstrual absorbent materials and provide adequate and safe WASH facilities to schools. Menstrual education should be strengthened in health extension packages to equip communities with accurate and practical information on menstruation. The importance of WASH facilities and affordable menstrual-absorbent materials should be closely linked to schoolgirls´ education and health.

**Recommendations:** based on the study findings; the WASH sectors should ensure that all schools have safe and adequate WASH facilities; WASH partners should provide funding and technical assistance to equip schools with WASH infrastructure; schools, local partners, and the private sector should also supply and distribute menstrual absorbent materials to schoolgirls; health extension workers should incorporate menstrual health education into their school outreach programs; the Ministry of Education should include menstrual health education in the school curriculum; the education sector must develop, implement, and enforce policies to address menstrual-related absenteeism; schools need to create a safe and inclusive environment, providing necessary facilities for schoolgirls; responsible sectors, such as education and health, should advocate jointly for policies and funding to enhance menstrual health management (MHM) in schools; furthermore, local government and community leaders, including health extension workers, should engage communities, religious leaders, local influencers, and traditional leaders to address sociocultural constraints. By implementing these measures, schools in study areas can help ensure that menstruation does not hinder educational opportunities for schoolgirls.

### What is known about this topic

- Menstrual hygiene is fundamental for all schoolgirls and women, but it is still an overlooked issue in most parts of the world;
- Menstruation affects schoolgirls´ school attendance;
- Shame and fear of menstruation matters are common in many cultures.

### What this study adds

- In the Northwestern part of Ethiopia, inadequate water, sanitation, and hygiene, socio-cultural constraints, teasing, fear, and inaccessibility of menstrual absorbent materials are factors for school absenteeism;
- Cultural and religious perceptions overshadow positive behavior in determining proper menstrual hygiene management, leading to school absenteeism, and should be integrated with any interventions;
- Health education on menstruation and menstrual hygiene management should target both schoolgirls and boys.
